# Associations between Physical Activity, Sitting Time, and Time Spent Outdoors with Mental Health during the First COVID-19 Lock Down in Austria

**DOI:** 10.3390/ijerph18179168

**Published:** 2021-08-31

**Authors:** Sandra Haider, Lee Smith, Lovro Markovic, Felipe B. Schuch, Kabir P. Sadarangani, Guillermo Felipe Lopez Sanchez, Rubén Lopez-Bueno, Alejandro Gil-Salmerón, Anita Rieder, Mark A. Tully, Lena Tschiderer, Lisa Seekircher, Peter Willeit, Igor Grabovac

**Affiliations:** 1 Department of Social and Preventive Medicine, Centre for Public Health, Medical University of Vienna, 1090 Vienna, Austria; sandra.a.haider@meduniwien.ac.at (S.H.); anita.rieder@meduniwien.ac.at (A.R.); igor.grabovac@meduniwien.ac.at (I.G.); 2 Centre for Health, Performance and Wellbeing, Anglia Ruskin University, Cambridge CB1 1PT, UK; Lee.Smith@aru.ac.uk; 3 Department of Physical Medicine, Rehabilitation and Occupational Medicine, Medical University of Vienna, 1090 Vienna, Austria; 4 Department of Sports Methods and Techniques, Federal University of Santa Maria, Santa Maria 97105-900, Brazil; felipe.schuch@ufsm.br; 5 Escuela de Kinesiología, Facultad de Salud y Odontología, Universidad Diego Portales, Santiago 8370179, Chile; kabir.sadarangani@gmail.com; 6 Department of Kinesiology, Universidad Autónoma de Chile, Santiago 7500912, Chile; 7 Vision and Eye Research Institute, School of Medicine, Faculty of Health, Education, Medicine and Social Care, Anglia Ruskin University, Cambridge CB1 1PT, UK; guillermo.lopez-sanchez@aru.ac.uk; 8 Department of Physical Medicine and Nursing, University of Zaragoza, 50009 Zaragoza, Spain; rlopezbu@unizar.es; 9 International Foundation for Integrated Care, Oxford OX2 6UD, UK; alejandrogilsalmeron@integratedcarefoundation.org; 10 Institute of Mental Health Sciences, School of Health Sciences, Ulster University, Newtownabbey BT37 0QB, UK; m.tully@ulster.ac.uk; 11 Clinical Epidemiology Team, Department of Neurology, Medical University of Innsbruck, 6020 Innsbruck, Austria; lena.tschiderer@i-med.ac.at (L.T.); lisa.seekircher@i-med.ac.at (L.S.); peter.willeit@i-med.ac.at (P.W.); 12 Department of Public Health and Primary Care, University of Cambridge, Cambridge CB1 8RN, UK

**Keywords:** COVID-19, physical activity, mental health, sitting time, Austria

## Abstract

Measures implemented to reduce the spread of SARS-CoV-2 have resulted in a decrease in physical activity (PA) while sedentary behaviour increased. The aim of the present study was to explore associations between PA and mental health in Austria during COVID-19 social restrictions. In this web-based cross-sectional study (April–May 2020) moderate-to-vigorous physical activity (MVPA), sitting time, and time spent outdoors were self-reported before and during self-isolation. Mental well-being was assessed with the Warwick-Edinburgh Mental Well-being Scale, and the Beck depression and anxiety inventories. The majority of the participants (*n* = 652) were female (72.4%), with a mean age of 36.0 years and a standard deviation (SD) of 14.4. Moreover, 76.5% took part in ≥30 min/day of MVPA, 53.5% sat ≥10 h/day, and 66.1% spent ≥60 min/day outdoors during self-isolation. Thirty-eight point five percent reported high mental well-being, 40.5% reported depressive symptoms, and 33.9% anxiety symptoms. Participating in higher levels of MVPA was associated with higher mental well-being (odds ratio = OR: 3.92; 95% confidence interval = 95%CI: 1.51–10.15), less depressive symptoms (OR: 0.44; 95%CI: 0.29–0.66) and anxiety symptoms (OR = 0.62; 95%CI: 0.41–0.94), and less loneliness (OR: 0.46; 95%CI: 0.31–0.69). Participants sitting <10 h/day had higher odds of mental well-being (OR: 3.58; 95%CI: 1.13–11.35). Comparable results were found for spending ≥60 min/day outdoors. Maintaining one’s MVPA levels was associated with higher mental well-being (OR = 8.61, 95%CI: 2.68–27.62). In conclusion, results show a positive association between PA, time spent outdoors and mental well-being during COVID-19 social restrictions. Interventions aiming to increase PA might mitigate negative effects of such restrictions.

## 1. Introduction

To reduce the spread and infection rate of SARS-CoV-2, the virus that causes coronavirus disease 2019 (COVID-19), the World Health Organisation (WHO) and various governments have enforced and are still enforcing public health interventions to promote physical distancing [1]. Additionally, individuals are encouraged to take up vaccination opportunities, wear masks, keep rooms well-ventilated, pay more attention to personal hygiene using hydroalcoholic gels, and go into self-isolation, whenever necessary. In Austria, the first “lockdown” measures were implemented on the 16 March 2020. The specific measures introduced by the Austrian government included limiting mobility, and individuals were advised to stay at home and limit their outings to the essentials such as buying food, going to the pharmacy or using medical services. Additionally, educational institutions and nonessential businesses (such as restaurants and bars, gyms, museums, theatres) were closed, and individuals were advised to work remotely (whenever possible) [2,3]. From 14 April onwards, some restrictions had been lifted. At the beginning of May, some of the sports facilities were also reopened and meetings of up to ten individuals in public spaces were permitted. Finally, on 15 May, museums as well as restaurants reopened with an extended curfew of 11 PM [2,3].

Studies from various countries have shown that during the COVID-19 pandemic physical activity (PA) decreased while sedentary behaviour increased. This was shown in various cross-sectional studies worldwide [4,5,6,7,8,9,10], and confirmed by several review articles [11,12]. Similar to international trends, studies from Austria have shown that during the COVID-19 pandemic, PA levels decreased by up to 48.9% [12,13]. Additionally, it was reported that mental health worsened in the general population during the pandemic [14,15]. For example, one study carried out in Austria reported an increase of severe depressive symptoms from 3% before the first COVID-19 lockdown to 6% during the COVID-19 pandemic [16].

Literature also suggests that the reduction in PA levels may have negative consequences on mental health [8], and is possibly associated with an increase in symptoms of anxiety and depression. Studies have demonstrated that PA is associated with lower anxiety and depression, while sedentary behaviour is associated with their increase [17,18,19]. Emerging data from the COVID-19 pandemic shows similar results [13,20,21,22], overall increasing the need to include PA as a health-promoting activity to reduce the mental health burden. It is noteworthy that, to the best of the authors’ knowledge, there are only few studies looking at the association between time spent outdoors and mental health. One of these studies, conducted in the USA, was able to show that individuals who increased or maintained the amount of time spent outdoors during the pandemic had lower level of stress and better positive mental health conditions compared to those who did not [23].

The purpose of the present study was to assess the association between PA and mental health among Austrians during the COVID-19 lock-down. In order to examine this association, also changes in PA levels and mental health status were investigated.

The results of this study may assist in creating appropriate strategies to mitigate mental health problems for future similar scenarios.

## 2. Material and Methods

### 2.1. Study Design and Recruitment

The present analysis is an evaluation of a cross-sectional study conducted in Austria. The survey was conducted online hosted on the Social Science Survey Platform (www.soscisurvey.de/COVID_studie, accessed on 16 April 2020) between 16 April and 16 May 2020, during the “first” lockdown enforced in Austria. Due to the given circumstances of physical distancing, participants were recruited via social media (e.g., Facebook, Twitter), printed media articles, and through word of mouth in existing researcher networks. The following inclusion criteria were applied: (i) age ≥18 years, (ii) currently living in Austria, and (iii) self-isolating due to the COVID-19 pandemic. Self-isolation was defined as staying at home and only leaving the household for essential activities such as working, food shopping or visiting the pharmacy or a health professional.

### 2.2. Ethical Statement

The study was approved by the Ethics Committee of the Medical University of Vienna on 14 April 2020 (number 1331/2020).

### 2.3. Study Variables

A total of 73 questions were included in the survey. In this study, variables associated with demographic data, PA, and psychological conditions were used for analysis.

#### 2.3.1. Demographic Data

The following data were collected: age, gender, marital status, net household income per year, employment status, current smoking status (yes, no), and alcohol intake (yes, no). If the participant indicated that they smoked or were drinking alcohol, they were asked a follow-up question on the number of cigarettes or alcoholic they consumed daily before and after the lockdown. Furthermore, previous mental and physical illnesses were surveyed by the questions “Have you ever been diagnosed with a mental illness by a doctor?” and “Have you ever been diagnosed with one or more chronic physical illnesses by a doctor?”, with suggested diseases and a multiple-choice option (e.g., obesity, high blood pressure, heart attack, angina pectoris and other coronary artery diseases, other diseases of the heart, osteoarthritis, chronic neck pain).

#### 2.3.2. Physical Activity Factors

All questions on PA were assessed through the use of the modified short form of the International Physical Activity Questionnaires (IPAQ) [24], in the validated German language version [25]. As such, the following questions were asked:

- Moderate-to-vigorous physical activity (MVPA)/day was assessed with the questions “Since you have been in home isolation, on average, how much time per day do you spend in moderate/vigorous physical activity?” Prior to analysis, data were checked for plausibility in the following ways: (1) if participants reported hours instead of minutes, or vice-versa, these values were transformed; (2) we have removed all entries reporting >16 h of MVPA [26]; (3) we have deleted unrealistic data. For logistic regression analyses, MVPA was dichotomized into ≥30 min/day and <30 min/day, following WHO recommendations [27].
- Sitting time/day was assessed with the question “Since you have been in home isolation, on average how much time per day do you spend sitting?” For logistic regression models, sitting time was treated as a dichotomous variable (<10 h sitting time/day; ≥10 h sitting time/day). This cut-off is based on the study of Gibson and colleagues [28].
- Time spent outdoors/day was asked with the question “Since you have been in home isolation, on average how much time per day do you spend outdoors?” Time spent outdoors was also dichotomised based on the median split of the present study (≥60 min/day; <60 min/day).

Identical questions were used to assess PA behaviour prior to self-isolation.

#### 2.3.3. Psychological Factors

Within this study mental health and psychological factors were assessed using four instruments:

- Warwick-Edinburgh Mental Well-being Scale short form (SWEMWBS): Mental well-being was categorized into four types, using the SWEMWBS [29]; probable depression (scores 7–17), possible depression (scores 18–20), average mental well-being (scores 21–27), and high mental well-being (scores 28–35) [30].
- Beck Depression Inventory (BDI): Originally, the level of depression is categorized into four types using the validated German version of BDI [31]; no depression (scores 0–9), mild (10–18), moderate (19–29), and severe depression (30–63) [32]. In our analysis individuals scoring between 10–63 points were described as exhibiting symptoms of depression.
- Beck Anxiety Inventory (BAI): Originally, the level of anxiety is categorized into four types using the validated German version of the BAI [33]; minimal anxiety (scores 0–7), mild anxiety (scores 8–15), moderate anxiety (scores 16–25), severe anxiety (scores 26–63) [34]. In our analysis, individuals with 8–63 points were described as having symptoms of anxiety.
- Three Item Loneliness Scale (TILS): Experienced loneliness was measured using the TILS [35]; lonely (3–5 points), not lonely (6–9 points).

### 2.4. Statistical Analysis

In analysing variables, we only used participants with given information on mental health and PA. In order to explore associations between MVPA, sitting time, time spent outdoors, and covariates indicating mental health outcomes, three logistic regression models were calculated, adjusted for age, gender, marital status, employment, household income, smoking and alcohol consumption, diagnosed chronic physical disease and mental illness, respectively. Logistic regression was also used to investigate if individuals who could maintain their MVPA behaviour during the pandemic were less affected by depression, anxiety, and loneliness. For this purpose, individuals were dichotomised into those who maintained the same levels or increased PA and those who decreased PA as compared to pre-pandemic times. These logistic regression models were adjusted in the same way as described above. All statistical analyses were performed with SPSS version 22.0 (BMI, Armonk, NY, USA).

## 3. Results

A total of 652 adults completed the survey. The sample predominantly consisted of women, and young adults, with a mean age of 36.0 years and a standard deviation (SD) of 14.4 (range: 18–92 years), who were single, divorced, widowed or separated. The majority were employed. The full details of the overall sample, stratified by the time spent in MVPA, sitting time, and time spent outdoors are shown in Table 1.

Change in PA patterns before and during isolation are shown in Table 2. As such MVPA and time spent outdoors decreased, while sitting time increased.

In terms of mental health and wellbeing (Table 3) the majority had average mental well-being. Based on the BDI score, 40.7% were classified as having depression. Additionally, 33.3% showed signs of anxiety. According to the TILS, nearly half of the participants indicated feeling lonely.

Further analysis showed no gender differences in mental well-being (*p* = 0.463), depression (*p* = 0.158), anxiety (*p* = 0.051), or loneliness (*p* = 0.293). There were also no significant differences in marital status regarding depression (*p* = 0.055) and loneliness (*p* = 0.102). However, a statistically significant difference was revealed for mental well-being (*p* = 0.030), whereas 43.7% of singles and 50.7% of participants in a partnership showed high mental well-being. Additionally, 69.7% of individuals in a partnership had no anxiety versus 60.6% of singles (*p* = 0.027).

The results of the logistic regression analyses are presented in Table 4. Participants who spent ≥30 min/day of MVPA were almost four times more likely to have high mental well-being as assessed by the SWEMWBS score compared to those who spent <30 min/day of MVPA. They were also less likely to be depressed, and to show symptoms of anxiety or loneliness. Our findings also indicate that those sitting <10 h/day were more likely to have high mental well-being. They were also less likely to be depressed, compared to individuals who sat ≥10 h/day. However, no statistically significant associations between sitting time and anxiety (*p* = 0.066) or loneliness (*p* = 0.676) were found.

Comparable results were seen with time spent outdoors. Individuals who spent ≥ 60 min/day outdoors had higher odds of having high mental well-being compared to individuals who spent <60 min/day outdoors. They were also more likely to have no depression compared to people who were outside <60 min/day. Again, no statistically significant associations with anxiety and loneliness were found.

The multivariable adjusted results of Table 5 revealed that individuals who managed to maintain their MVPA level during self-isolation were more likely to have high mental well-being, no depression, and no symptoms of anxiety, and no loneliness compared to those individuals who reduced their MVPA behaviour.

## 4. Discussion

The findings of this study show a decline in the time respondents spent in MVPA and outdoors, with an increases in time spent sitting. Participants reporting sufficient min/day of MVPA were significantly more likely to report high mental well-being, to exhibit no symptoms of depression or anxiety, and were more likely not to report feeling lonely. Furthermore, sitting < 10 h/day was positively associated with mental well-being and negatively with depression, showing no statistically significant associations with anxiety or loneliness. Comparable results were found for spending ≥ 60 min/day outdoors. Additionally, our results revealed that maintaining MVPA levels during self-isolation could be important for sustaining mental well-being.

Our findings suggest that higher levels of MVPA are associated with reduced depressive symptoms, and are in line with previous studies conducted in various countries across the globe [20,36,37,38,39]. For instance, this was also confirmed in a sample of 8425 adults living in UK, Ireland, New Zealand and Australia [21]. Additionally, the observed relationship between sitting time and depressive symptoms in our study is comparable to findings by Schuch and colleagues, who reported that individuals spending ≥ 10 h/day on sedentary behaviour were more likely to present with depressive symptoms, but not anxiety [20]. Referencing Hallgren et al. [40], Schuch et al. postulated that sedentary periods of more than 10 h/day are needed to increase anxiety [20]. Additionally, as more than half of the participants were employed, we assume that longer sitting time might also be a result of time spent working from home. Thus, additional PA as a result of commuting to work was not a possibility. However, it is important to note that the association between PA and mental health, particularly depression, is bidirectional and no cause or consequence can be established.

Additionally, the correlation between maintaining pre-pandemic MVPA levels and psychological well-being is supported by findings from an American survey that included 3052 participants and indicated that not reaching PA guidelines was related to depression, loneliness, and stress [41]. Additionally, a study among 3971 identical twins in the US has shown that a decrease in PA was associated with higher stress and anxiety levels, with the association between anxiety and PA remaining significant after controlling for genetic and environmental factors [42].

Looking at the level of MVPA, we observed an overall decrease after self-isolation measures. When comparing these data to Austrian data collected before the pandemic, the percentage of individuals doing regular PA was in the present data set higher, as in the data of the Eurobarometer, showing that 30% of the Austrian adults are physically active on a regular basis [43]. However, this high amount of MVPA is in accordance with the results reported by Smith and colleagues [4], indicating that 75.0% of participants had sufficient MVPA during social distancing, with a mean of 94.0 (SD: 119.1) min/day. In this survey, conducted in the UK, MVPA was assessed in the same way. The same examination done in Brazil, indicated that 53.4% of the participants did ≥30 min/day of MVPA [20]. In summary, it can be said that the level of MVPA was high in all of these studies, which might be due to the participating population (young, well educated). Additionally, an over-estimation of PA might be possible [44,45].

The fact that 67.1% of the population was outdoors for ≥60 min/day was also unexpectedly high, when bearing in mind that from March to April, people were only allowed to leave their homes to go to work, buy groceries, help other people, or go for a walk. Considering the characteristics of the majority of the participants, this result may be driven from the younger adults. However, this is only an assumption. When one looks at the results in more detail, they revealed that being outdoors for ≥60 min/day is associated with a higher mental well-being and a lower chance for being depressive. In this context it has also be mentioned that especially in urban areas the enforced restrictions on mobility and the closed indoor fitness centres leads to limited PA opportunities [46]. Additionally, the study by Geng and colleagues, found that stay-at-home restrictions are negatively associated with park visits [47].

Taking a closer look at the psychological factors the present study observed marginally higher scores in BAI and lower BDI scores in comparison to the above-mentioned Brazil population, with comparable socioeconomic factors [20]. Comparing our data to another Austrian COVID-19 survey, we found considerably higher prevalence in depression, measuring 21% of depressive and 19% of anxiety symptoms [38]. Differences might be justified by different measurement tools. Notably, unlike Niehnaus [48], we did not observe any gender differences in well-being, depression, or anxiety. Concerning loneliness, we observed comparable results to an UK sample, with 41% of our sample being classified as feeling lonely [49].

Based on the present results and the available literature, the following implications can be derived: the association between low PA levels and rising mental health issues is relevant for public health and should be taken into account when considering any type of restriction measures. Additionally, as insufficient PA is a known key risk factor for cardiovascular disease, hypertension, diabetes type 2, and prostate and colon cancer [50,51], interventions aiming at increasing MVPA levels and decreasing time spent sitting should be promoted as much as reasonably possible. Considering that PA levels of most individuals decreased during the pandemic, especially in people with chronic illness [52], it seems of special interest to all public health stakeholders that conditions should be created enabling individuals to maintain their usual MVPA levels. Nevertheless, given the positive effects of PA on mental health outcomes, measures should be taken to promote outdoor activities and opportunities for outdoor exercises. Considering the development of the pandemic since March 2020, it would be of interest to analyse changes in PA behaviour and mental health over time as well as between country differences.

Some limitations have to be mentioned. Due to the recruitment method through social media (Facebook, Instagram, Twitter), which was necessary under this particular situation, it can be assumed that there was a selection bias. As such, the sample was mostly composed of well-educated women and young adults, whereas participants of lower education and higher age are underrepresented in our sample. Therefore, we may assume that the reported PA levels are higher in comparison with the general Austrian population during the COVID-19 lockdown. Additionally, people who experienced some mental health difficulties or have psychological illness might have been more inclined to participate in the study as they felt the topic was more relevant. This may lead to an overestimation of depression and anxiety. Another limitation is that data was self-reported, which can lead to an overestimation of PA [44,45]. Finally, the cross-sectional design of the study does not permit any conclusion concerning directionality in the relationship between PA and psychological factors.

## 5. Conclusions

In conclusion, the present results show a positive association between PA and mental well-being during the infection control measures, in a web-based nonrepresentative sample of the Austrian population. Results also show that maintaining MVPA behaviour seems to be important to sustain mental well-being. Therefore, interventions to promote and support higher MVPA levels and a decrease in sitting time during self-isolation should be supported according to the possibilities in order to maintain mental well-being.

## Figures and Tables

**Table 1:** Participants’ characteristics.

Variables	No (%)	≥30 MVPA min/Day	<10 Sitting Hours/Day	≥60 min Outdoors/Day
	Overall	498 (76.5)	415 (53.5)	514 (66.1)
Age				
18–34 years	373 (56.2)	76.3	46.7	61.1
35–64 years	256 (38.6)	75.1	59.6	71.5
≥65 years	35 (5.3)	88.6	80.0	80.0
Gender				
Female	481 (72.4)	78.9	55.5	68.3
Male	176 (26.5)	70.7	49.1	60.9
Nonbinary, transfer, or intersex	7 (1.1)	57.1	28.6	42.9
Marital status				
Married or registered partnership	167 (25.2)	80.5	65.1	76.0
In a relationship but not married	231 (34.8)	77.6	47.7	62.2
Single, divorced, widowed, separated	266 (40.1)	72.4	51.8	63.3
Net household income per year				
<16,000 €	165 (25.9)	80.2	53.4	64.8
16,000–26,000 €	136 (20.5)	78.0	53.4	67.6
26,001–41,000 €	169 (25.5)	74.4	50.9	67.3
41,001–60,000 €	120 (18.1)	77.1	55.1	63.0
>60,000 €	74 (11.1)	69.0	57.5	68.5
Employment status				
Employed	390 (58.7)	73.5	51.8	63.8
Student	200 (30.1)	80.3	48.4	66.7
Homemaker	9 (1.4)	100	100	88.9
Retired, not able to work	49 (7.4)	83.7	79.6	81.6
Unemployed	16 (2.4)	66.7	50.0	56.3
Current smoking status, yes	112 (16.9)	72.7	47.7	65.2
Alcohol intake, yes	399 (60.1)	76.8	51.9	64.4
Diagnosed chronic physical illness, no	269 (40.5)	74.0	44.5	63.5
Diagnosed mental illness, no	513 (77.3)	75.6	54.5	59.3

Categorical variables are shown as percentages (%). No = number; MVPA = moderate to vigorous physical activity.

**Table 2:** Change in physical activity patterns before and during isolation.

Physical Activity	Before	Isolation
≥30 min/min of MVPA	89.0%	76.3%
Median sitting h/day (IQR)	8 (5–9)	9 (6–12)
Median time spent outdoors min/day (IQR)	120 (90–180)	60 (30–180)

MVPA = moderate to vigorous physical activity; IQR = interquartile range.

**Table 3:** Psychological factors.

	m (SD); Median (IQR); %	≥30 MVPA min/Day	<10 Sitting Hours/Day	≥60 min Outdoors/Day
**Warwick-Edinburgh Mental Well-being Scale Short form**	25.7 (4.3)	26.0 (4.2)	26.5 (4.1)	26.1 (4.2)
Probable depression (7–17 points)	25 (3.9)	56.5	20.0	44.4
Possible depression (18–20 points)	57 (8.8)	62.5	49.1	63.2
Average mental well-being (21–27 points)	314 (48.5)	76.4	51.3	64.0
High mental well-being (28–35 points)	252 (38.9)	82.4	60.7	72.6
**Beck Depression Inventory**	7.5 (3–13)	7 (3–12)	6 (2–12)	7 (3–12)
No depression (0–9 points)	381 (59.3)	82.0	60.1	70.9
Depression (10–63 points)	261 (40.7)	69.8	44.4	59.8
**Beck Anxiety Inventory**	7.4 (1–10)	4 (1–9)	4 (1–9)	4 (1–9)
No anxiety (0–7 points)	424 (66.7)	79.5	57.3	70.0
Anxiety (8–63 points)	212 (33.3)	71.5	46.2	58.5
**Loneliness Scale**				
Lonely (3–5 points)	342 (52.5)	82.3	56.4	70.5
Not lonely (6–9 points)	309 (47.5)	70.3	50.2	61.5

Descriptive data for continuous variables are shown in mean (m) and standard deviation (SD), if normally distributed, or in median and interquartile range (IQR), if data are non-normally distributed. Categorical variables are shown as percentages. No = Number; MVPA = moderate to vigorous physical activity.

**Table 4:** Association between physical activity and psychological factors.

	≥30 Versus <30 MVPA min/Day			<10 Versus ≥10 Sitting Hours/Day			≥60 Versus <60 min Outside/Day		
	OR	95% CI	*p*	OR	95% CI	*p*	OR	95% CI	*p*
**Warwick-Edinburgh Mental Well-being Scale Short form** (**SWEMWBS)**									
Probable depression (7–17 points)	1.00			1.00			1.00		
Possible depression (18–20 points)	1.98	0.43–3.36	0.732	3.58	1.13–11.35	0.031	1.93	0.73–5.15	0.187
Average mental well-being (21–27 points)	2.61	1.06–6.46	0.038	3.48	1.22–9.92	0.020	1.93	0.83.4.48	0.128
High mental well-being (28–35 points)	3.92	1.51–10.15	0.005	4.61	1.58–13.43	0.005	2.44	1.01–5.86	0.047
**Beck Depression Inventory (BDI)**									
No depression (0–9 points)	1.00			1.00			1.00		
Depression (10–63 points)	0.44	0.29–0.66	<0.001	0.60	0.43–0.85	0.004	0.68	0.48.0.96	0.030
**Beck Anxiety Inventory (BAI)**									
No anxiety (0–7 points)	1.00			1.00			1.00		
Anxiety (8–63 points)	0.62	0.41–0.94	0.024	0.71	0.50–1.02	0.066	0.70	0.49–1.02	0.060
**Loneliness Scale (TILS)**									
Not lonely (6–9 points)	1.00			1.00			1.00		
Lonely (3–5 points)	0.46	0.31–0.69	<0.001	0.93	0.67–1.30	0.676	0.74	0.52–1.04	0.080

Results from the logistic regression analyses (dependent variables: ≥30 min of MVPA/day <10 sitting h/day and ≥60 min outdoors/day) are presented as odds ratios (ORs) with their 95% confidence intervals (CIs), respectively. Results are adjusted for age, gender, marital status, employment, household income, current smoking, current alcohol consumption, diagnosed chronic physical or mental illnesses. MVPA = moderate to vigorous physical activity. In all analyses only data with complete data were included. For the SWEMWBS 669 cases were included; for the BDI 663 cases, for the BAI 657, and for the TILS 672 individuals were used.

**Table 5:** Association between change in physical activity and psychological factors. Results are adjusted for age, gender, marital status, employment, household income, current smoking, current alcohol consumption, diagnosed chronic physical or mental illnesses. MVPA= moderate to vigorous physical activity.

	Equal or More MVPA		
	OR	95% CI	*p*
**Warwick-Edinburgh Mental Well-being Scale Short form**			
Probable depression (≤17 points)	1.00		
Possible depression (18–20 points)	3.24	0.93–11.22	0.064
Average mental well-being (21–27 points)	4.20	1.34–13.15	0.014
High mental well-being (28–35 points)	8.61	2.68–27.62	<0.001
**Beck Depression Inventory**			
Depression (10–63 points)	1.00		
No depression (0–9 points)	2.57	1.80–3.67	<0.001
**Beck Anxiety Inventory**			
Anxiety (8–63 points)	1.00		
No anxiety (0–7 points)	2.01	1.39–2.90	<0.001
**Loneliness Scale**			
Lonely (3–5 points)	1.00		
Not lonely (6–9 points)	2.62	1.85–3.70	<0.001

## Data Availability

The data presented in this study are available on request from the corresponding author.
